# Supplementary material for: Temperature and quantum anharmonic lattice effects on stability and superconductivity in lutetium trihydride
Source: Nat Commun. 2024 Jan 10;15:441. doi: 10.1038/s41467-023-44326-4 (PMC10781996; doi:10.1038/s41467-023-44326-4)
Supplement: Supplementary file 1 — Supplementary Information [file 41467_2023_44326_MOESM1_ESM.pdf]

Supplementary information
for
'Temperature and quantum anharmonic lattice effects on stability and superconductivity in lutetium trihydride'

Roman Lucrezi,[1] Pedro P. Ferreira,[1,2] Markus Aichhorn,[1] and Christoph Heil[1,*]

[1] *Institute of Theoretical and Computational Physics, Graz University of Technology, NAWI Graz, 8010 Graz, Austria*

[2] *Universidade de São Paulo, Escola de Engenharia de Lorena, DEMAR, 12612-550, Lorena, Brazil*

(Dated: December 4, 2023)

* Corresponding author; christoph.heil@tugraz.at

I. SUPPLEMENTARY METHODS

Supplementary Method 1. Details on the SSCHA calculations

The SSCHA phonon dispersions shown in the main manuscript are obtained from the positional free-energy Hessians in the *bubble approximation*, i.e. including only the second and third order derivatives of the Born-Oppenheimer potential energy surface $E_{\mathrm{el}}[\mathbf{R}]$ in

$$\frac{\partial^2 \mathcal{F}}{\partial \mathcal{R} \partial \mathcal{R}} = \mathbf{\Phi}^{(2)} + \mathbf{\Phi}^{(3)} \mathbf{\Lambda} \left[\mathbf{1} - \mathbf{\Phi}^{(4)} \mathbf{\Lambda} \right]^{-1} \mathbf{\Phi}^{(3)}, \qquad \text{where } \Phi^{(n)}_{a_i \dots a_n} = \left\langle \frac{\partial^n E_{\mathrm{el}}[\mathbf{R}]}{\partial \mathcal{R}_{a_1} \dots \partial \mathcal{R}_{a_n}} \right\rangle_{\tilde{\rho}_{\Phi, \mathcal{R}}}, \tag{1}$$

and $\mathbf{\Lambda}$ is a fourth-order tensor containing the eigenvalues and eigenvectors of the auxiliary system [1]. In Supplementary Figure 1, we show the validity of this approximation by comparing the phonon dispersion obtained with second, third, and fourth order derivatives of $E_{\mathrm{el}}[\mathbf{R}]$, and find that the fourth order contributions add only minor changes to the phonon dispersions.

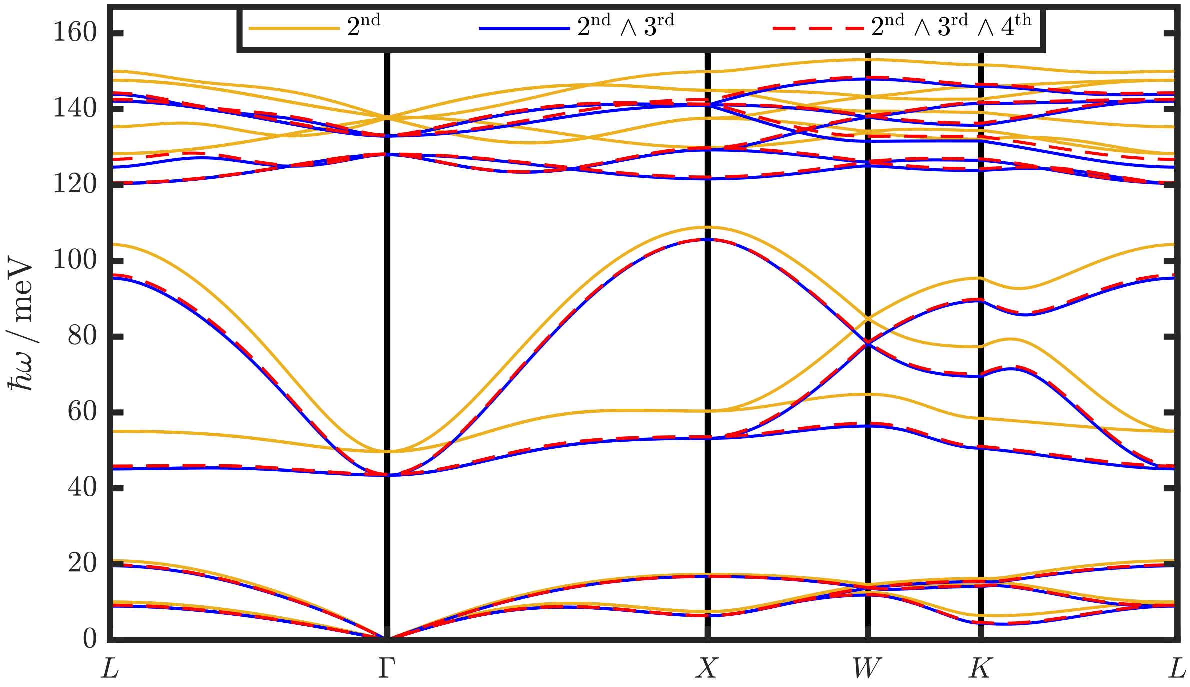

Supplementary Figure 1: Second order (auxiliary matrices), third order (*bubble approximation*), and fourth order contributions to the phonon dispersions obtained from the positional free energy Hessian at $p = 2.8\,\mathrm{GPa}$ and $T = 300\,\mathrm{K}$.

II. SUPPLEMENTARY DISCUSSIONS

Supplementary Discussion 1. Additional SSCHA phonon dispersions

In Supplementary Fig. 2 we show the full energy range of the phonon dispersions as a function of pressure shown in Fig. 2 in the main text. In Supplementary Fig. 3 we show the low-energy range of the phonon dispersions for all considered $p-T$ combinations, which highlights the $p-T$ dependence of the structural instabilities at $\mathbf{q}=X$ and $\mathbf{q}=K$.

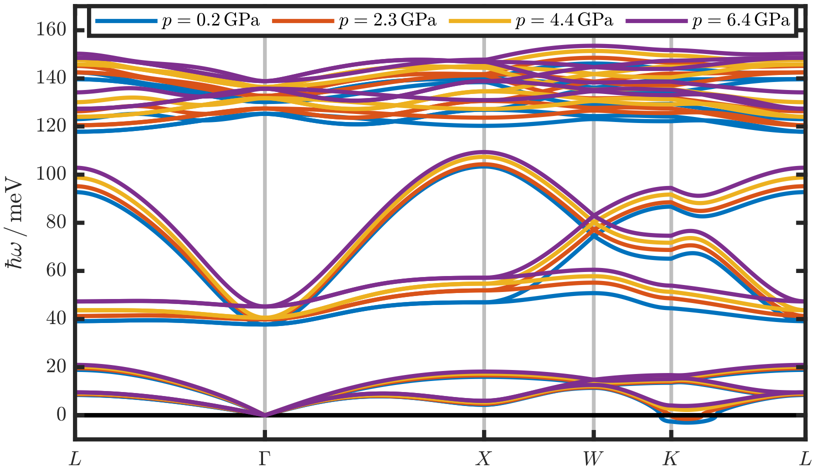

Supplementary Figure 2: Full energy range of the phonon dispersions as a function of pressure at a fixed temperature of 150 K in the 2×2×2 supercell.

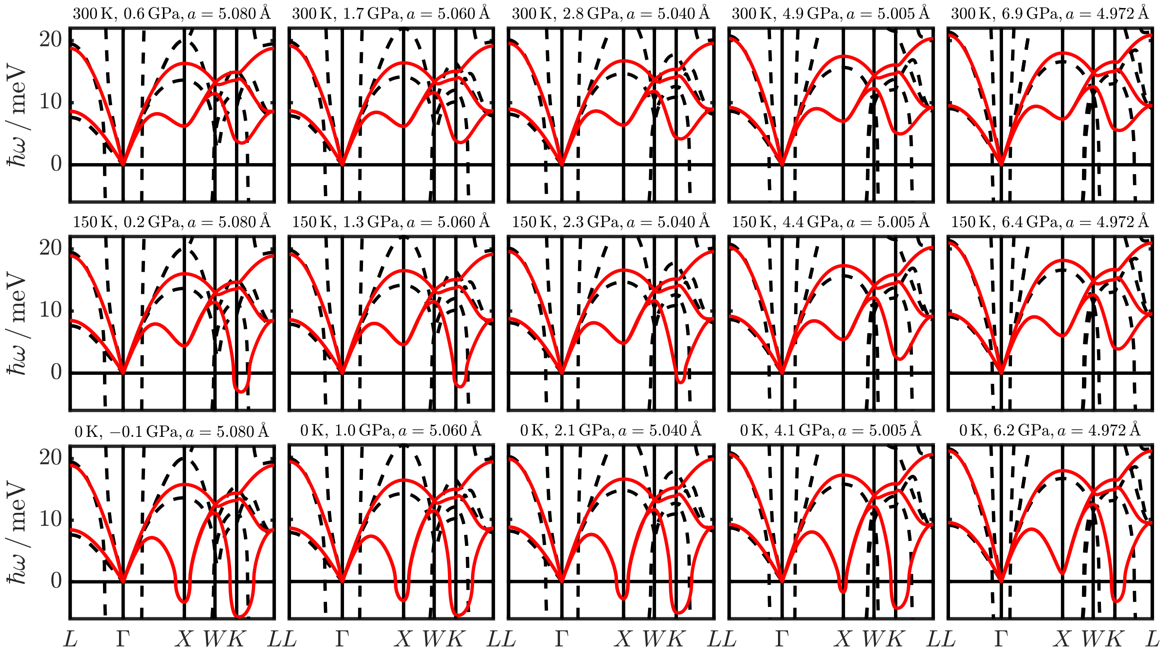

Supplementary Figure 3: Low-energy range of the phonon dispersion for all considered $p-T$ combinations in the 2×2×2 supercell. The harmonic phonon dispersion for the corresponding lattice constant a at $T=0\,\mathrm{K}$ are shown as dashed lines.

Supplementary Discussion 2. Supercell convergence and functional forms

We want to stress here again that the all results and figures in the main text are obtained via DFT calculations employing ONCV-PBE pseudopotentials in $2 \times 2 \times 2$ supercells. In the following section, we test and show results for different combinations of exchange-correlation functional forms, pseudopotentials, and supercells – either DFT or MTP based.

We tested three different combinations of pseudopotentials (PP) and functional forms of the exchange-correlation functional, namely (*i*) optimized norm-conserving Vanderbilt (ONCV) PPs in the PBE parametrization, (*ii*) projector augmented wave (PAW) PPs in the PBE parametrization, and (*iii*) ONCV PPs in the PZ parametrization. In the following, the labels "-PZ" and "-PBE" refer to both, pseudopotential generation and exchange-correlation functional form in the actual DFT calculation. The ONCV-PZ pseudopotentials are generated using the ONCVPSP code and match the settings of the SG15 collection, described in [2, 3]. For our final calculations, we chose the ONCV-PBE setting, as the obtained unit-cell (uc) lattice constant $a_{\text{ONCV-PBE}}$ is closest to the experimental value $a_{\text{exp}} = (5.12 \pm 0.02)$ Å at ambient conditions [4], once temperature and quantum anharmonic effects are included. The various lattice constants at $T = 300$ K are summarized in Tab. 1. We find an almost constant shift of ~ 5 GPa between the DFT pressure p_{DFT} and the SSCHA pressure p at $T = 300$ K for all tested settings (see Supplementary Fig. 4), allowing for a comparison between p_{DFT} and $p(300\,\text{K})$ based on the ONCV-PBE SSCHA calculations. The numerical settings for the DFT calculations with ONCV-PZ and PAW-PBE are the same as described in the Methods section in the main text for ONCV-PBE, with an additional kinetic energy cutoff for the charge density of 600 Ry for PAW-PBE.

Supplementary Table 1: Unit-cell (uc) lattice constants at $T = 300$ K and various pressures for the settings (*i*) to (*iii*). Lattice constant a in Å, DFT pressure p_{DFT} in GPa, and pressure p in GPa within the constant-volume SSCHA calculations within ONCV-PBE.

p_{DFT}	$p(300\,\text{K})$	$a_{\text{ONCV-PBE}}$	$a_{\text{PAW-PBE}}$	$a_{\text{ONCV-PZ}}$
-4.0	0.6	5.080	5.042	4.944
-2.0	2.8	5.040	5.007	4.914
0.0	4.9	5.005	4.974	4.886
2.0	6.9	4.972	4.944	4.860

In Supplementary Fig. 4, we compare the influence of the employed PPs and the functional form of the exchange-correlation functional on the converged SSCHA phonon dispersions in the $2\times2\times2$ supercell. We performed three separate SSCHA runs at 300 K with a fixed lattice constant of $a = 5.040$ Å, resulting in different pressures p. The acoustic modes are almost independent of the employed settings, the main differences appear on the path $\Gamma - K$, which also shows an intricate convergence behaviour, as shown in Supplementary Fig. 5 to 8. The optical modes are noticeably softened in ONCV-PZ, where they are slightly hardened in PAW-PBE for strongly anharmonic modes at Γ, X, and L which is expected as very small details have large influence there. We point out that considering the substantially lower pressure value obtained with ONCV-PZ at fixed a, the phonon frequencies match the PBE results well. In Supplementary Figure 4(b), we show the effect of balancing the pressure values in an ONCV-PZ calculation with a reduced lattice constant of $a = 4.914$ Å.

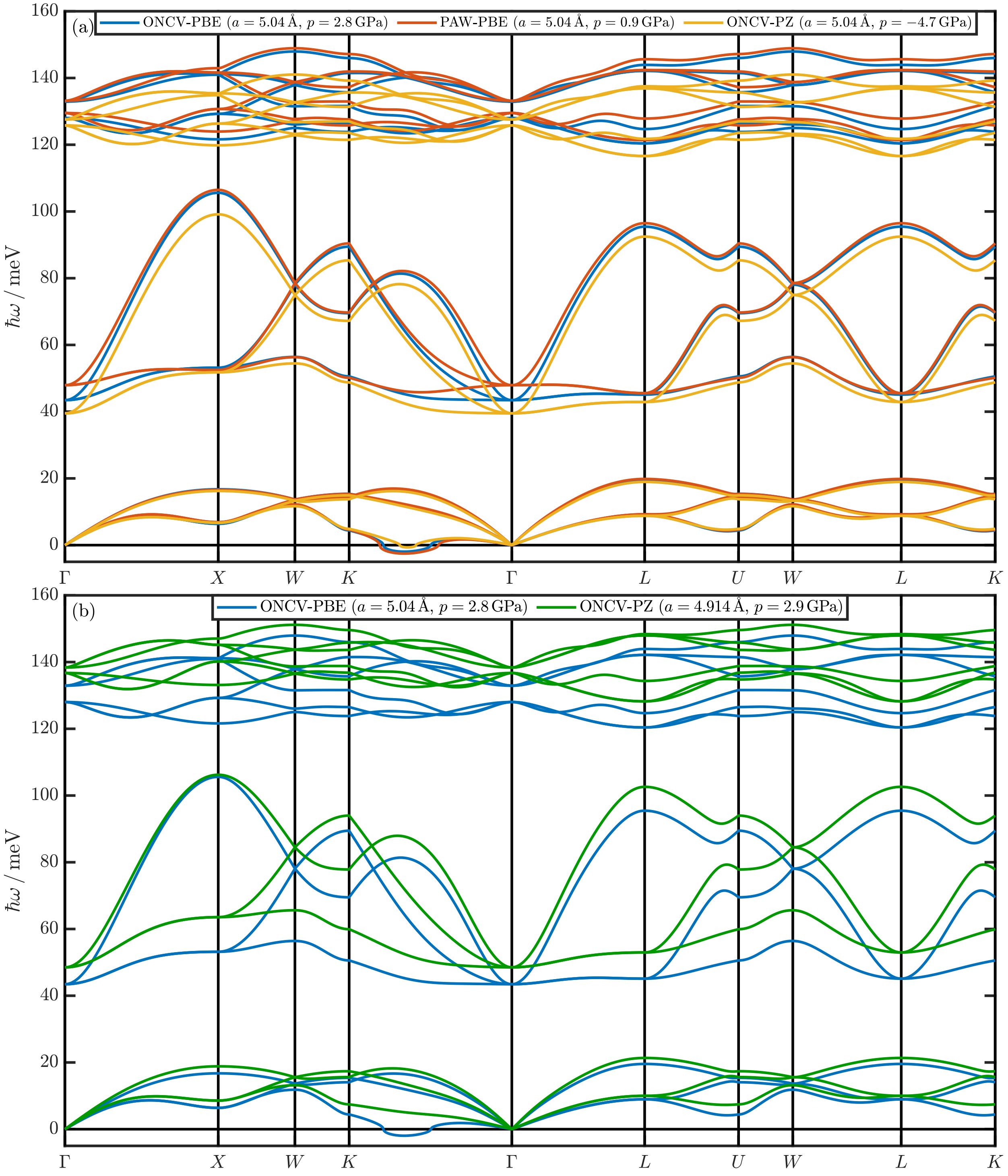

Supplementary Figure 4: (a) SSCHA phonon dispersion obtained with different functional and PP form in the 2×2×2 supercell at $T = 300$ K and $a = 5.04$ Å. The pressures obtained in SSCHA are indicated in the legend. The corresponding DFT pressures are -2.0, -3.9, and -9.5 GPa for ONCV-PBE, PAW-PBE, and ONCV-PZ, respectively. (b) Comparison of ONCV-PBE (blue) and ONCV-PZ with a similar calculated pressure (green).

SSCHA is a supercell-based method and the convergence has to be checked with respect to the supercell size. In order to deal with the enormous computational costs of DFT calculations with several hundreds to thousands of atoms, we employed machine-learned moment tensor potentials (MTP) [5, 6] to calculate total energies, forces, and stresses in higher supercells with DFT accuracy. Using the approach described in our recent work [7], we trained different MTPs on sets consisting of 45 SSCHA individuals at $T = 300\,\mathrm{K}$, 15 each in 2×2×2, 3×3×3, and 4×4×4 supercells (containing 32, 108, and 256 atoms, respectively). Choosing a functional form of level 26, eight radial basis functions, $R_{\mathrm{cut}} = 5.0\,\text{Å}$ and $R_{\min} = 1.3 - 1.5\,\text{Å}$, we obtain root-mean-squared errors (RMSE) on the total energy of $\sim 1\,\mathrm{meV/atom}$, $\sim 40\,\mathrm{meV/Å}$ for the force components, and $\sim 0.3\,\mathrm{GPa}$ for the diagonal stress tensor components, when validated on all individuals in the corresponding DFT SSCHA calculations in 2×2×2 supercells. The exact RMSE values and a graphical representation of the validation are shown in Supplementary Fig. 14. In Supplementary Fig. 5, we show the convergence of the low-energy phonon dispersion with respect to the employed supercell size, calculated with an MTP trained on ONCV-PZ calculations.

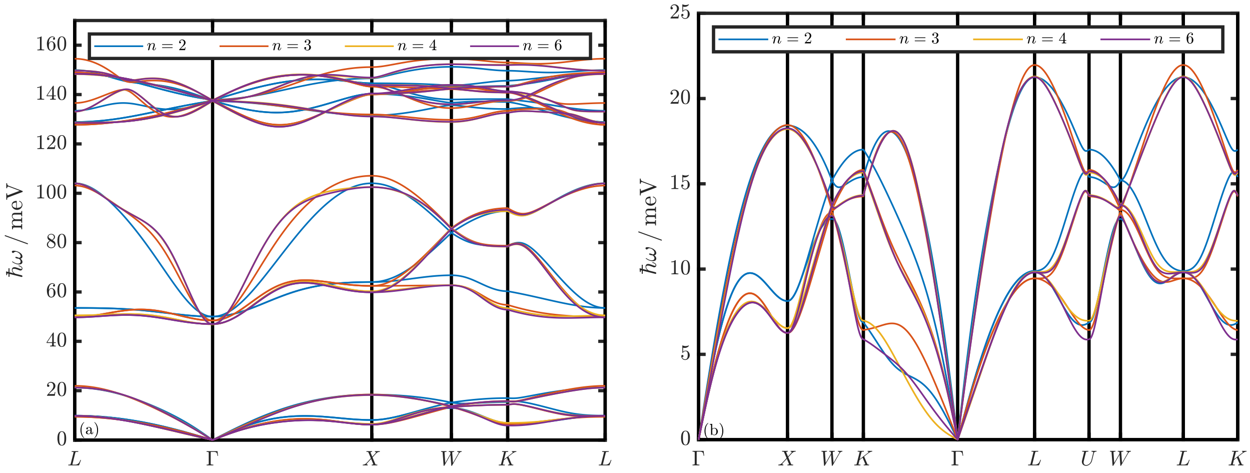

Supplementary Figure 5: Phonon dispersions for supercell sizes $n \times n \times n$ ($n = 2, 3, 4, 6$, containing 32, 108, 256, and 864 atoms, respectively). (a) Full energy range along the same high-symmetry Brillouin-zone path as in the main text, and (b) Low-energy range along an extended path including explicitly $\Gamma - K$. The SSCHA calculations are preformed at $T = 300\,\mathrm{K}$, yielding a pressure of $p = 2.9\,\mathrm{GPa}$. The SSCHA individuals are evaluated with an MTP trained on ONCV-PZ calculations with a uc lattice constant $a = 4.914\,\text{Å}$.

In Supplementary Fig. 6, we show the convergence of the phonon dispersion with respect to the employed supercell size for different pressures, calculated with an MTP trained on ONCV-PBE calculations. Higher supercell calculations for $p = 2.8\,\mathrm{GPa}$ suggest an instability at $\mathbf{q} = X$ that is not captured in the $2{\times}2{\times}2$ supercell, but disappears for slightly higher temperatures than $300\,\mathrm{K}$ (see Supplementary Fig. 7). The instability on the path $\Gamma - K$ is persistent with higher temperatures (see Supplementary Fig. 7). Overall, these two instabilities show a peculiar convergence trend with respect to the supercell size for $p = 2.8\,\mathrm{GPa}$ that does not unambiguously answer the question of dynamic (in)stability. Due to memory size limitations, even higher supercell calculations are not feasible with our current computational resources (e.g. an $n = 8$ supercell requires 5.1 TB of RAM on a single shared-memory node for the calculation of the Hessian matrices within the *bubble approximation*, as three double-precision arrays with $(3 \cdot n^3 \cdot <\text{number of atoms in uc}>)^3$ elements are needed). For $p = 6.9\,\mathrm{GPa}$, the mode at $\mathbf{q} = X$ is slightly softened for supercells with $n > 2$, but stable at $300\,\mathrm{K}$. The path $\Gamma - K$ shows a similar behaviour as for $p = 2.8\,\mathrm{GPa}$, and stability is not definitively ruled out either (see also Supplementary Fig. 8). For $p = 11.1\,\mathrm{GPa}$, we obtain positive phonon frequencies in the whole Brillouin zone and find a reasonable convergence behaviour with respect to the supercell size.

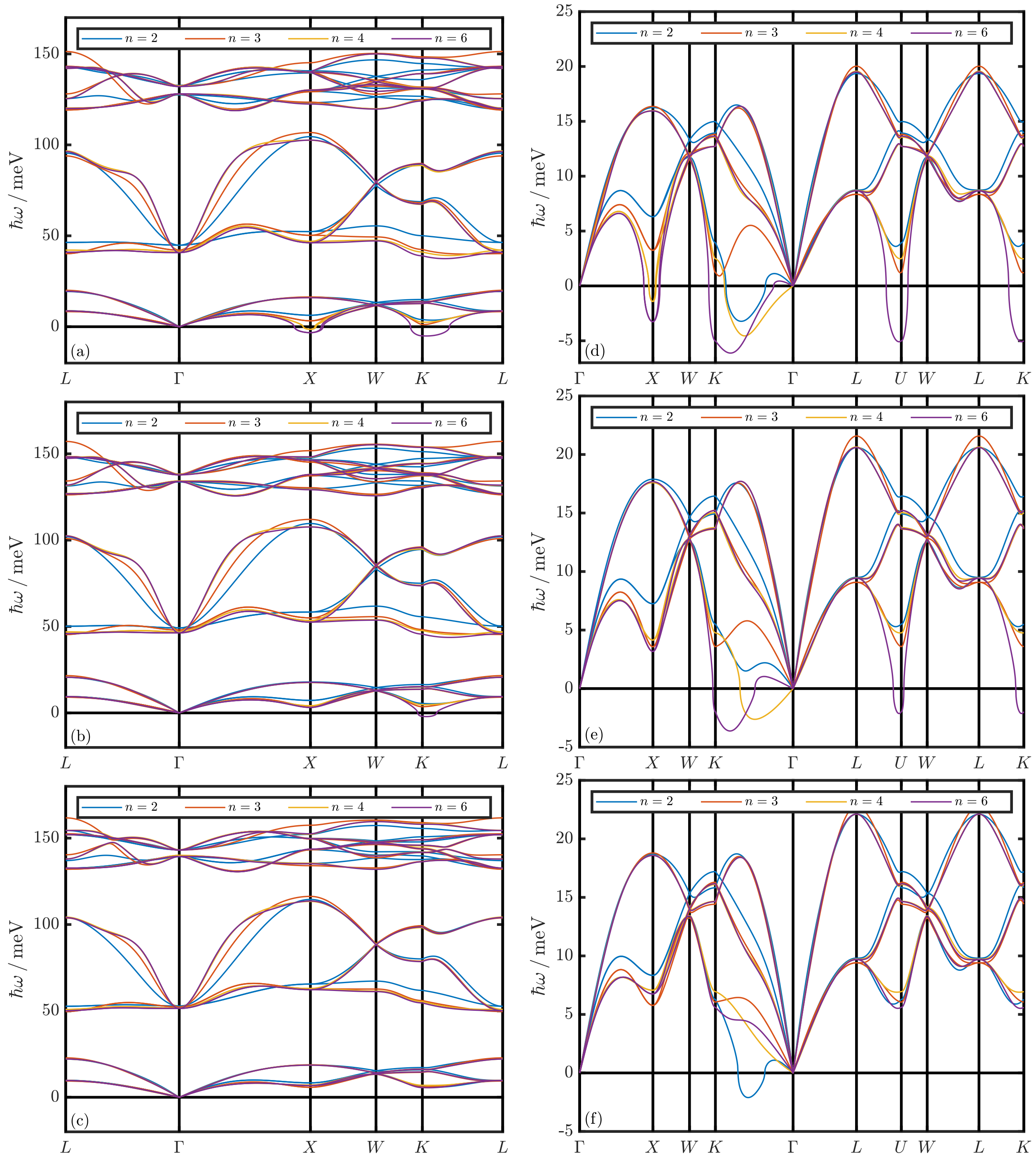

Supplementary Figure 6: Supercell convergence at $T = 300\,\mathrm{K}$ and $p = 2.8, 6.9$, and $11.1\,\mathrm{GPa}$. (a)-(c): full energy range along the same high-symmetry Brillouin-zone path as in the main text, (d)-(f): low-energy details along an extended path including explicitly $\Gamma - K$. The SSCHA individuals are evaluated with different MTPs trained on ONCV-PBE calculations with uc lattice constants 5.040, 4.972, and 4.915 Å, respectively.

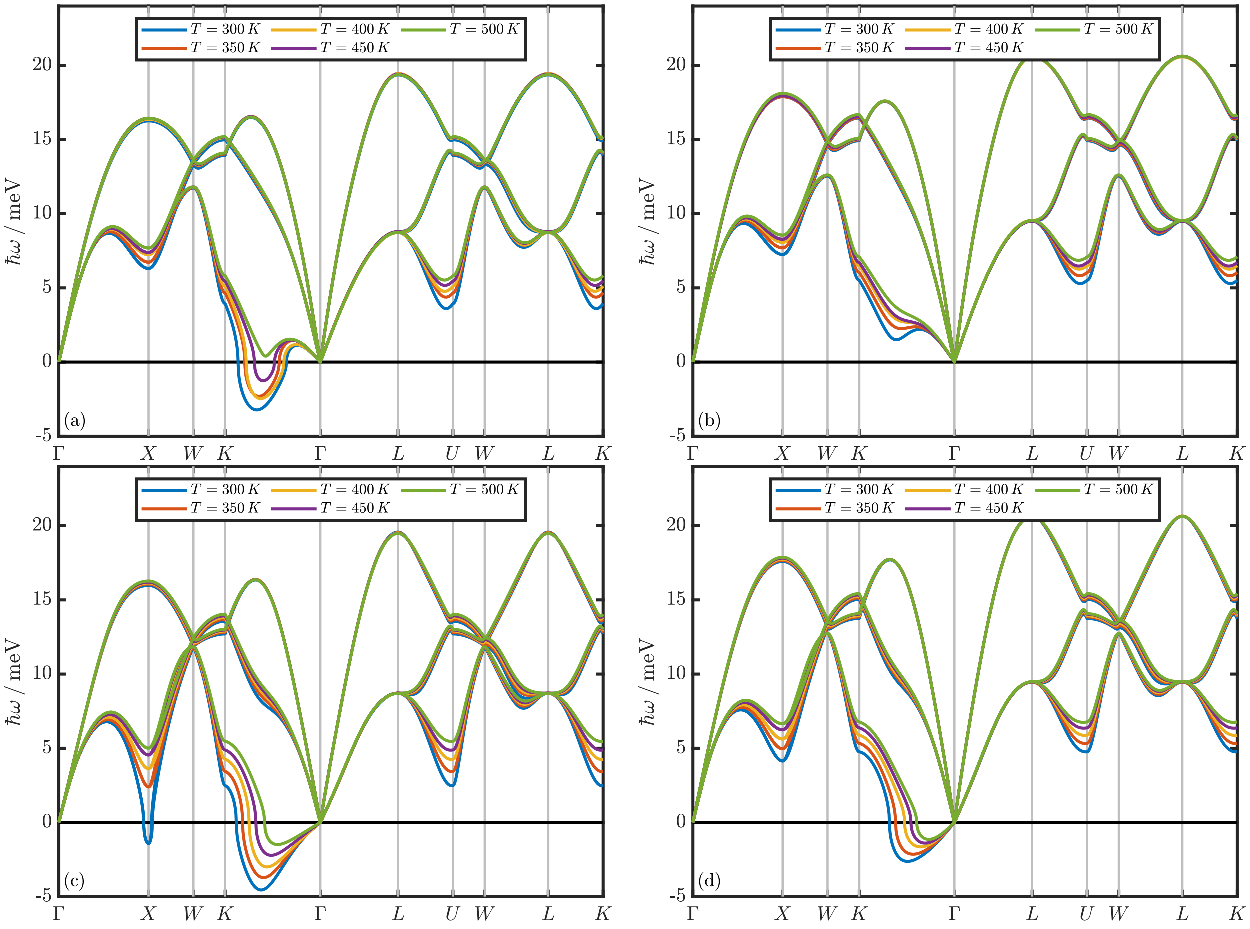

Supplementary Figure 7: Low-energy phonon dispersions obtained in (a)-(b) 2×2×2 and (c)-(d) 4×4×4 supercells at $T \geq 300\,\mathrm{K}$ and pressures above (a),(c) 2.8 GPa and (b),(d) 6.9 GPa. The indicated pressures refer to the value obtained at $T = 300\,\mathrm{K}$. For a fixed lattice constant, higher temperatures lead to a higher pressure. The SSCHA individuals are evaluated with different MTPs trained on ONCV-PBE calculations with uc lattice constants (a),(c) 5.040 Å, and (b),(d) 4.972 Å.

In Supplementary Fig. 8, we further demonstrate the fragility of the convergence behaviour of the phonon frequencies on the path $\Gamma - K$. For $p = 6.9\,\mathrm{GPa}$, we trained an MTP on a different random training set at an elevated temperature of $T = 500\,\mathrm{K}$, and recalculated the phonon dispersion for various temperatures. Considering the stochastic nature of the SSCHA, the two MTPs yield identical phonon dispersions, except for two modes on the path $\Gamma - K$, and, interestingly, around L. In particular on $\Gamma - K$, one MTP predicts an instability (see Supplementary Fig. 7), while the other predicts stable phonon dispersions (except for interpolation artifacts close to Γ).

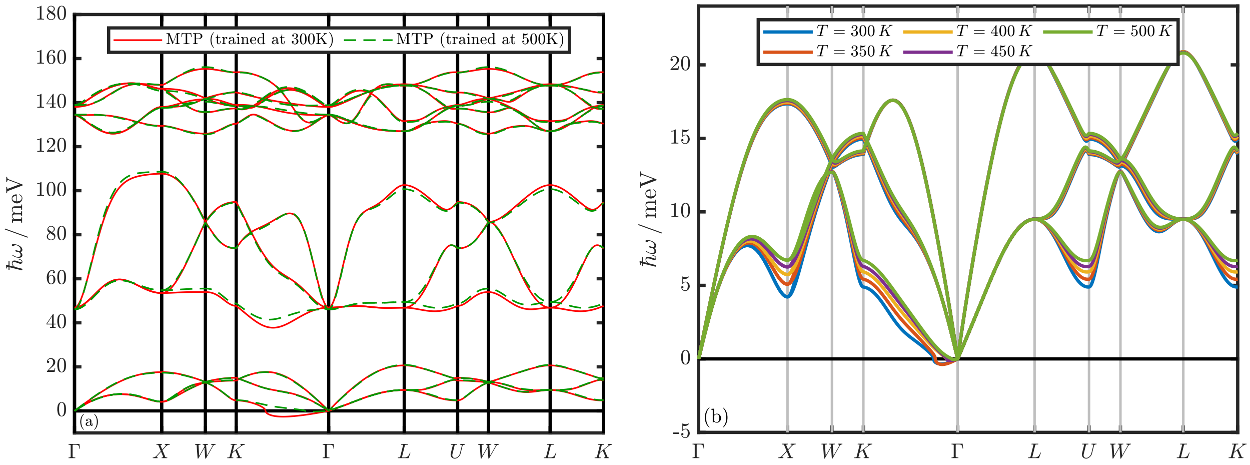

Supplementary Figure 8: Comparison of the phonon dispersions at 6.9 GPa and 300 K obtained from SSCHA calculations in 4×4×4 supercells with two different MTPs (a), and at various temperatures (b) obtained with an MTP trained at 500 K (compare with Supplementary Fig. 7). The MTPs are trained on ONCV-PBE calculations with a unit-cell lattice constant of 4.972 Å.

III. SUPPLEMENTARY FIGURES

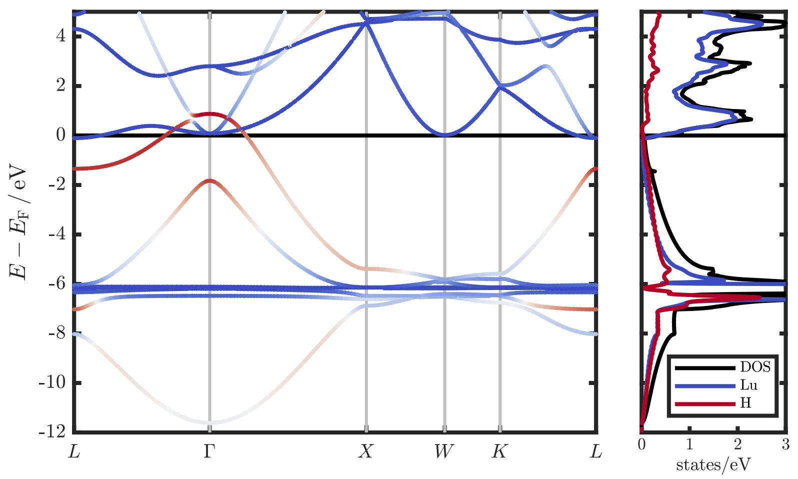

Supplementary Figure 9: Electronic dispersions with H contributions indicated as colored markers and (partial) DOS for a wider energy window around the Fermi level. The almost dispersion-less lutetium f states around -6 eV lead to a high peak of about 130 states/eV in the DOS, which is not shown.

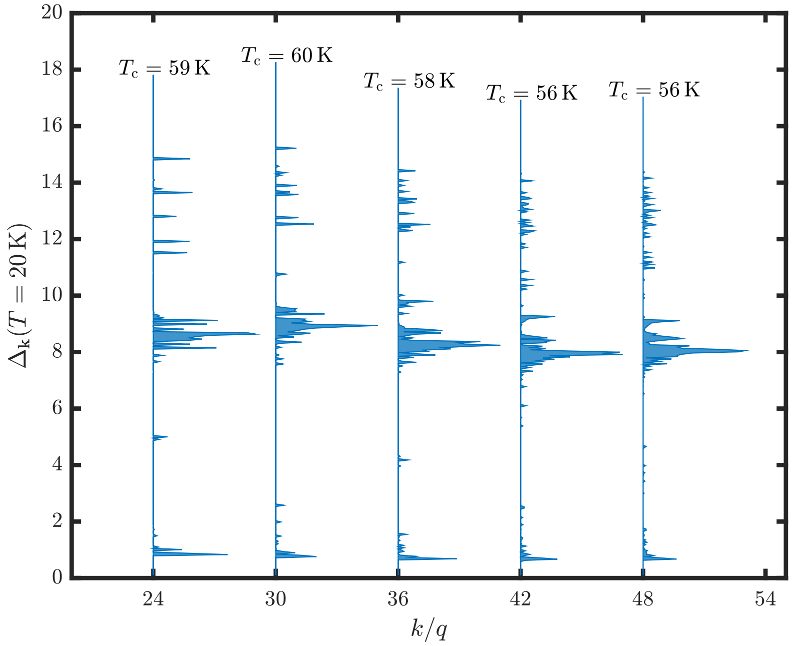

Supplementary Figure 10: Convergence of the anisotropic superconducting gap function $\Delta_{n\mathbf{k}}$ shown in Supplementary Fig. 11 at $T = 20\,\mathrm{K}$ with respect to the fine $\mathbf{k}$- and $\mathbf{q}$-grids $k \times k \times k$ and $q \times q \times q$, respectively. The numerical artifacts of low- and high-energy values of $\Delta_{n\mathbf{k}}$ are persistent with increasingly dense grids, the resulting critical temperature T_c varies only within $4\,\mathrm{K}$.

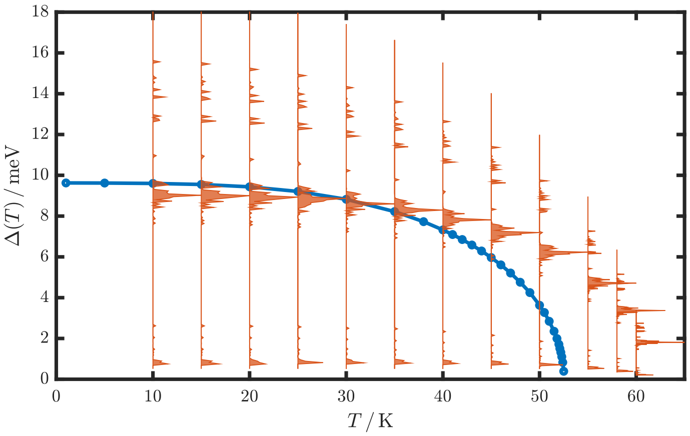

Supplementary Figure 11: Superconducting gap function as a function of temperature in undoped LuH_3. The Migdal-Eliashberg (ME) equations are solved for a lattice constant of $a = 5.040$ Å, corresponding to $p = 2.8$ GPa at $T = 300$ K, and using the corresponding SSCHA phonon dispersions obtained in the 2×2×2 supercell and interpolated onto a 6×6×6 **q**-grid. Within EPW, the electronic and phononic states are interpolated onto fine **k**- and **q**-grids of 30×30×30. Blue dots (shaded red areas) show the solution to the isotropic (anisotropic) ME equations. The low-energy (< 2 meV) and high-energy (> 12 meV) values of the anisotropic gap function $\Delta_{n\mathbf{k}}$ are numerical artifacts of **k**-points that are not part of the Fermi surface and are persistent with denser **k**- and **q**-grids (see Supplementary Fig. 10).

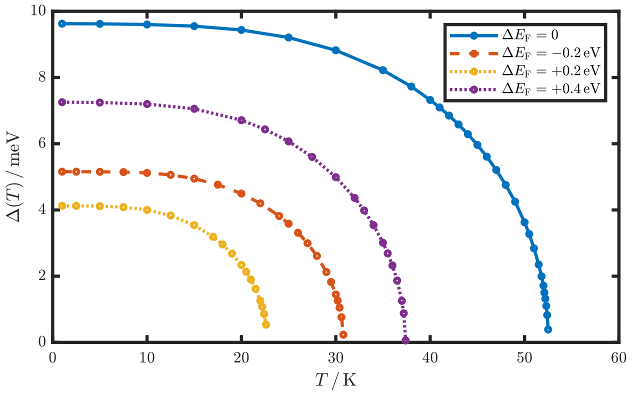

Supplementary Figure 12: Isotropic superconducting gap function as a function of temperature for different rigid band shifts. The solid blue line represents the unshifted case, the dashed red line a negative shift in the Fermi energy, and the dotted lines positive shifts. For positive shifts, the T_c from anisotropic solutions is $\sim 10\,K$ higher than the isotropic shown here. For the negative shift, the anisotropic calculations do not converge. Due to the extremely low density of states at the Fermi energy, an extremely dense **k**-grid is needed to generate explicit points at the Fermi energy.

Supplementary Table 2: Overview of the performed EPW calculations and obtained critical temperatures T_c within isotropic (anisotropic) Migdal-Eliashberg equations, denoted by IME (AME). The calculations are performed for three different lattice constants and two SSCHA temperatures, corresponding to the pressures given in Tab. I in the main text. The Fermi energy shift ΔE used in the rigid-band approximation is given in eV, the critical temperature T_c in K. The dynamical matrices are obtained from the SSCHA calculations on a 2×2×2 supercell interpolated onto a 6×6×6 **q**-grid, as described in the main text. For the cases denoted by a dagger (†), the interpolation to a dense **q**-grid leads to imaginary phonon frequencies along $\Gamma - K$, which are set to zero in the EPW calculations.

	phonons at 150 K						phonons at 300 K					
	2.3 GPa†		4.4 GPa†		6.4 GPa		2.8 GPa		4.9 GPa		6.9 GPa	
ΔE	T_c^{IME}	T_c^{AME}	T_c^{IME}	T_c^{AME}	T_c^{IME}	T_c^{AME}	T_c^{IME}	T_c^{AME}	T_c^{IME}	T_c^{AME}	T_c^{IME}	T_c^{AME}
-0.2	36	10	37	12	33	14	31	6	30	9	29	10
0.0	56	64	56	61	52	54	53	60	51	57	43	50
0.2	24	36	20	29	16	25	23	33	18	27	15	23
0.4	39	49	30	38	24	33	38	48	27	37	23	32

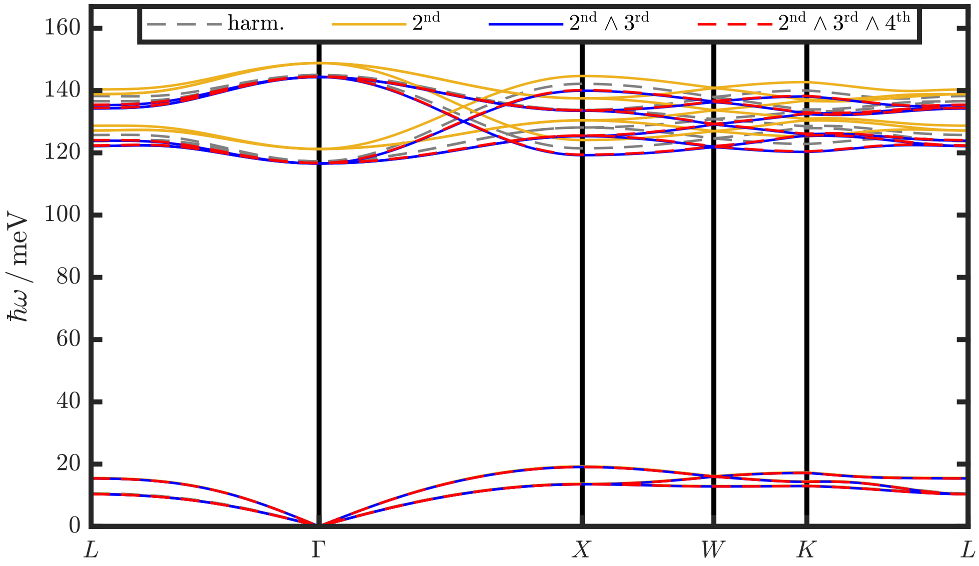

Supplementary Figure 13: Different contributions to the SSCHA phonon dispersion for LuH_2 at 2.4 GPa and 0 K, obtained in a 2×2×2 supercell. The SSCHA individuals are evaluated with ONCV-PBE calculations with uc lattice constants of 5.018 Å. See Supplementary Fig. 1 for details on the different contributions. In an additional SSCHA calculation at 300 K, we only find a minor change in the optical modes of LuH_2 in the order of 1 to 2 meV.

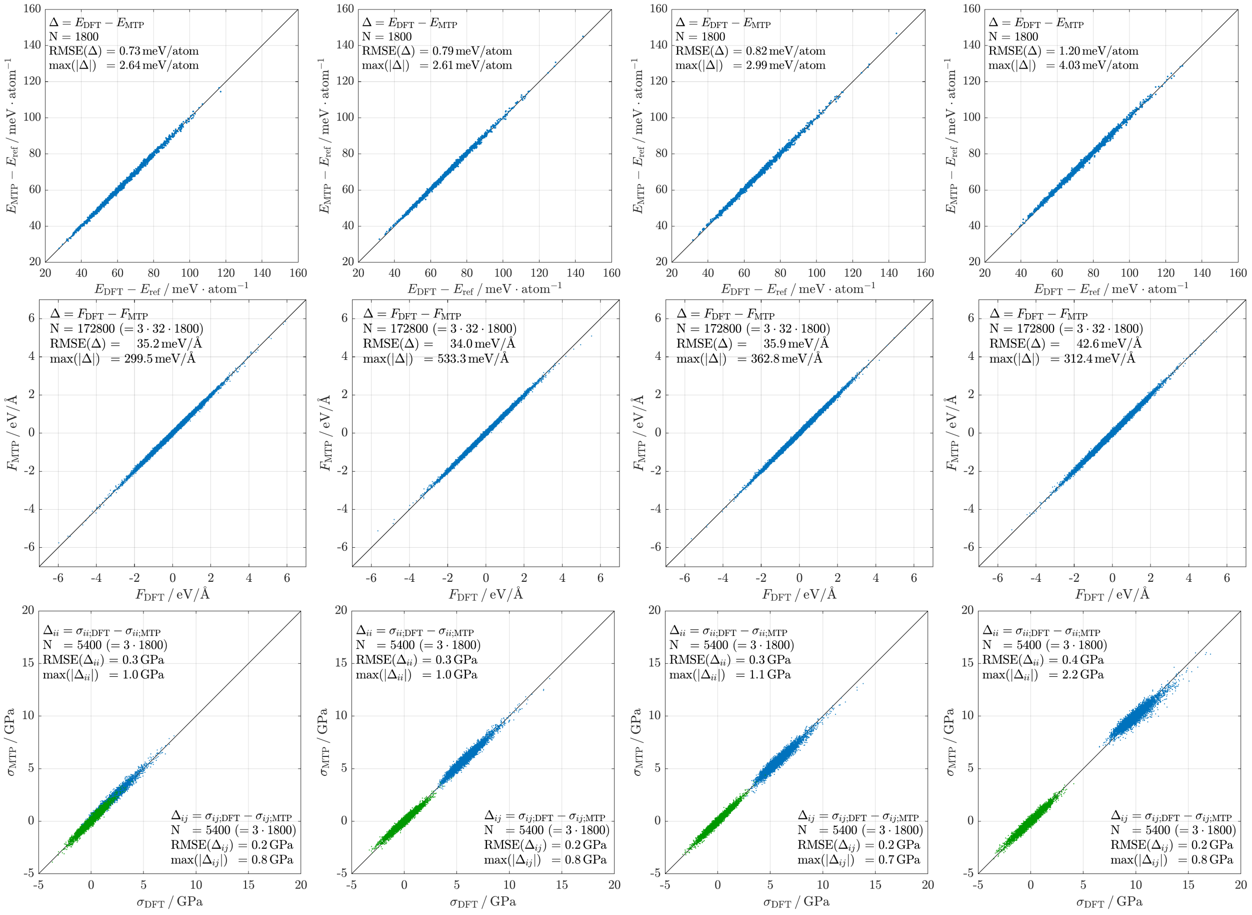

(a) 5.040 Å (b) 4.972 Å (c) 4.972 Å (500 K) (d) 4.915 Å

Supplementary Figure 14: MTP validation for the predicted energies E_{MTP}, force components F_{MTP}, and stress tensor components σ_{MTP} of the individuals generated in the DFT SSCHA calculations for lattice constants of (a) 5.040 Å, (b)-(c) 4.972 Å, and (d) 4.915 Å. The MTPs corresponding to (a)-(d) are used to obtain the SSCHA results shown in Supplementary Fig. 6 and 7, the MTPs corresponding to (b) and (c) are used to obtain the SSCHA results shown in Supplementary Fig. 8. Each subpanel shows the MTP prediction versus the corresponding DFT values. The energies are plotted with respect to the DFT total energy of the undisplaced structure E_{ref} with the indicated lattice constant. The diagonal elements of the stress tensor σ_{ii} are plotted as blue dots, the off-diagonal elements $\sigma_{i\neq j}$ as green dots. The diagonal black line represents the reference for a perfect prediction.

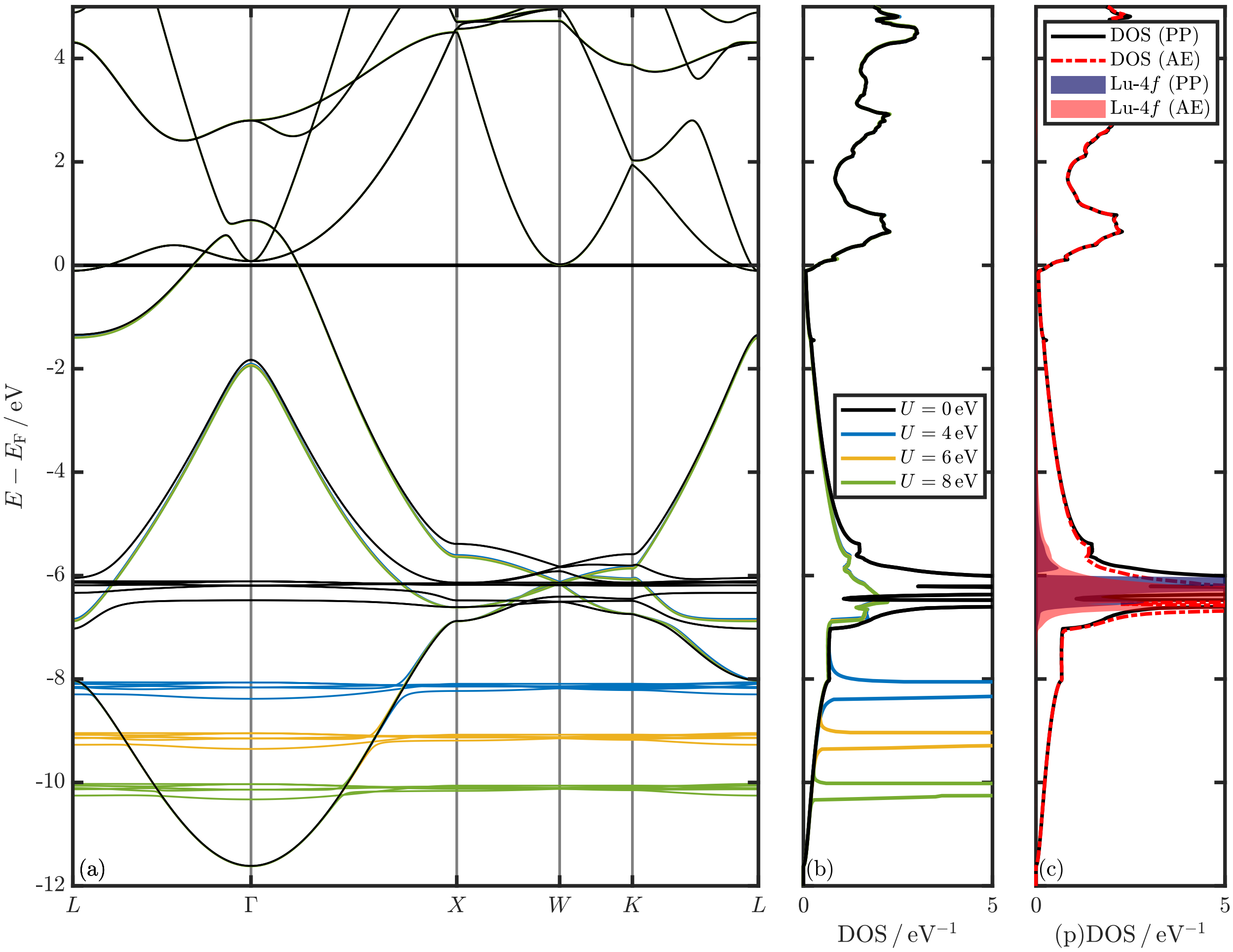

Supplementary Figure 15: (a) Electronic dispersion and (b) corresponding DOS for LuH_3 with a lattice constant of $a = 5.040$ Å for a regular DFT calculation within the ONCV-PBE setting (black line) and for DFT+U calculations with $U = 4$, 6, and 8 eV for the Lu-f states (coloured lines). The different lines lie on top of each other for energy values around and above the Fermi energy E_F. (c) DOS (lines) and partial (p) DOS (shaded areas) for the Lu-4f states with $U = 0$ eV calculated with pseudopotentials (PP) within the ONCV-PBE setting and in an all-electron (AE) calculation with Wien2k. For the latter, the size of the LAPW basis set was determined by $R \cdot K_\mathrm{max} = 7.0$ and muffin-tin radii were chosen as $2.50\,a_0$ and $1.43\,a_0$ for Lu and H, respectively. We used PBE as xc-potential and 1059 irreducible $\mathbf{k}$-points were used for the BZ sampling.

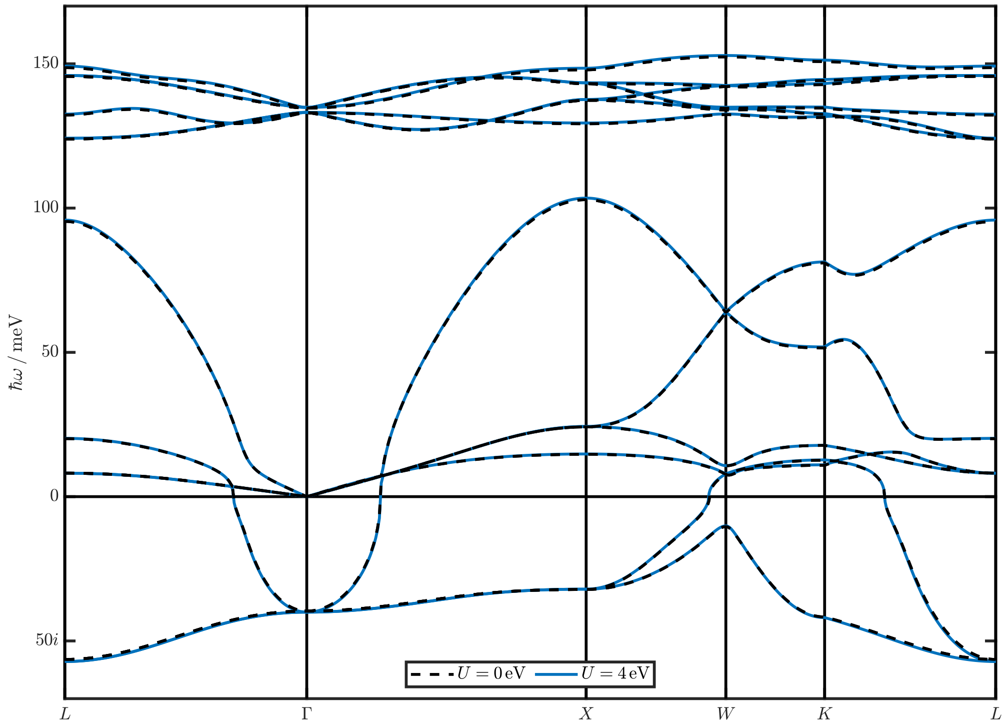

Supplementary Figure 16: Harmonic phonon dispersions on a 2×2×2 **q**-grid for LuH_3 with a lattice constant of $a = 5.040$ Å for a regular DFPT calculation within the ONCV-PBE setting (dashed black line) and for a DFPT+U calculation with $U = 4$ eV for the Lu-f states (solid blue line). The maximum differences in the two dispersion are < 0.5 meV.

SUPPLEMENTARY REFERENCES

[1] R. Bianco, I. Errea, L. Paulatto, M. Calandra, and F. Mauri, Second-order structural phase transitions, free energy curvature, and temperature-dependent anharmonic phonons in the self-consistent harmonic approximation: Theory and stochastic implementation, Phys. Rev. B **96**, 014111 (2017).

[2] D. R. Hamann, Optimized norm-conserving Vanderbilt pseudopotentials, Phys. Rev. B **88**, 085117 (2013).

[3] M. Schlipf and F. Gygi, Optimization algorithm for the generation of ONCV pseudopotentials, Comput. Phys. Commun. **196**, 36 (2015).

[4] T. Palasyuk and M. Tkacz, Pressure-induced structural phase transition in rare-earth trihydrides. part i.(GdH_3, HoH_3, LuH_3), Solid state communications **133**, 481 (2005).

[5] A. V. Shapeev, Moment tensor potentials: A class of systematically improvable interatomic potentials, Multiscale Modeling & Simulation **14**, 1153 (2016).

[6] K. Gubaev, E. V. Podryabinkin, G. L. Hart, and A. V. Shapeev, Accelerating high-throughput searches for new alloys with active learning of interatomic potentials, Computational Materials Science **156**, 148 (2019).

[7] R. Lucrezi, E. Kogler, S. Di Cataldo, M. Aichhorn, L. Boeri, and C. Heil, Quantum lattice dynamics and their importance in ternary superhydride clathrates, Communications Physics **6**, 298 (2023).
